# Patterns of Radiotherapy Utilization for Lung Cancer Patients with Brain Metastases: A Population-based Analysis

**DOI:** 10.7759/cureus.5591

**Published:** 2019-09-07

**Authors:** Roel Schlijper, Ian M Fraser, Jacqueline Regan, Shilo Lefresne, Cheryl Ho, Robert A Olson

**Affiliations:** 1 Radiation Oncology, British Columbia Cancer Agency, Prince George, CAN; 2 Radiation Oncology, University of British Columbia, Vancouver, CAN

**Keywords:** brain, neoplasm metastasis, cranial irradiation, decision-making, mortality, risk

## Abstract

Introduction: Brain metastases occur in 15%-20% of lung cancer patients. Recently, studies have suggested that whole-brain radiotherapy (WBRT) may not prolong survival for a subset of patients, and is associated with significant side-effects. Furthermore, it is hypothesized that radiotherapy is often given near the end-of-life when the potential for benefit is minimal. Therefore, this study investigates how frequently radiotherapy for brain metastases is given near the end-of-life in a population-based cohort.

Materials and methods: All lung cancer patients who received radiotherapy in British Columbia for brain metastases in 2014-2015 were identified. Patient and treatment characteristics were collected and analyzed to assess associations with death within 90 days of first radiation treatment.

Results: In total, 740 patients were identified, with a total of 826 courses of brain radiation. The 90-day mortality rate was 40% (n=330). Multivariable analysis demonstrated higher odds for age (odds ratio (OR) = 1.04, 95% confidence interval (CI) 1.02-1.05), Eastern Cooperative Oncology Group (ECOG) performance score of 2 or higher (OR = 1.59, 95% CI 1.09-2.31) and squamous cell carcinoma (OR = 2.10, 95% CI 1.13-3.90) and lower odds for initial systemic therapy (OR = 0.48, 95% CI 0.34-0.68), more than five fractions of radiotherapy (OR = 0.25, 95% CI 0.16-0.39) and stereotactic radiation (OR = 0.29, 95% CI 0.13-0.65).

Conclusion: In our population-based study, WBRT is given in 86% of radiotherapy courses for brain metastases from lung cancer. Of these patients, 40% received treatment near the end-of-life. We identified several factors associated with shortened survival. Using these factors and already established prognostic tools, WBRT utilization should be decreased in the future, improving individualized treatment for patients with brain metastases from lung cancer.

## Introduction

Brain metastases are common in lung cancer, occurring in approximately 15%-20% of patients [1-2]. There are several treatment options for patients with brain metastases, of which whole-brain radiotherapy (WBRT) is the most common. WBRT can cause several side-effects, particularly in the first three months following treatment, such as fatigue, nausea, hair loss, and scalp dermatitis, leading to a negative impact on the quality of life [3-4]. Alternatives to WBRT are stereotactic radiotherapy (SRT) either in one fraction (stereotactic radiosurgery (SRS)) or multiple fractions (fractionated stereotactic radiotherapy (SFRT)), surgical resection, systemic therapy alone, and best supportive care.

The optimal management of patients with brain metastases from lung cancer is controversial with variations in international practice [5]. Many studies have investigated SRT as compared to or in addition to WBRT in select patient populations to improve outcome and/or minimize toxicity [6-8]. However, selection criteria for the different radiotherapy options are a topic of debate. Prior studies have shown that patients with brain metastases from lung cancer, treated with WBRT, have a median overall survival of approximately 2.5-4.5 months [9-11]. The Quality of Life after Treatment for Brain Metastases (QUARTZ) trial identified no significant difference in survival or quality of life between patients treated with WBRT and optimal supportive care vs. optimal supportive care alone. There was a nonsignificant trend towards improved survival for patients younger than 60, with Karnofsky performance status of 70+ and a controlled primary non-small cell lung cancer (NSCLC) [4]. Less than 20% of their patients had at least one of these factors, therefore a major proportion of patients in the trial that received WBRT experienced adverse-effects at the end of their lives, without improvement in the quality of life or survival. How these findings apply to patients with brain metastases in the general lung cancer population is of utmost concern.

Prognostic tools like the recursive partitioning analysis (RPA) classes and the Graded Prognostic Assessment (GPA-mol) have been developed for patients with brain metastases in an effort to predict survival [11-13]. The RPA categorizes patients into three classes, based on age, Karnofsky performance status (KPS) and control of extracranial disease. GPA-mol categorizes patients using age, KPS, extracranial disease, number of brain metastases and presence or absence of epidermal growth factor receptor (EGFR) and anaplastic lymphoma kinase (ALK) mutations. Patients in the worst groups, RPA-class III and GPA-mol score of ≤1.0 had a median survival of 2 and 5-7 months respectively and may not benefit from WBRT [11-12,14]. In contrast, patients in the best groups, like GPA-mol score of ≥3.5 had a median survival of almost four years [12]. These patients may benefit from the maximized local control and limited toxicity of SRT. Improving the selection of patients who would benefit from radiotherapy for brain metastases is important in improving quality of life and outcomes, both in patients with good prognosis and for those that are near the end of life.

Population-based patterns of care and outcome for lung cancer patients with brain metastases are not well described in the literature. The aim of this population-based study is to describe patterns of radiotherapy utilization for treatment of brain metastases in lung cancer patients. Furthermore, we aim to identify patients that died 90 days of start of radiotherapy. We hypothesize that the majority of patients were treated with WBRT with only a subgroup of patients receiving SRT. Furthermore, we hypothesize that in our population, 20%-30% of patients receiving brain radiotherapy died within 90 days of radiation treatment.

## Materials and methods

All lung cancer patients in British Columbia (BC), Canada, who received any radiation treatment for brain metastases between January 1, 2014 and December 31, 2015, were identified using the BC Cancer electronic medical record. Patient and treatment characteristics were collected. Radiation treatment was categorized as WBRT, partial brain radiotherapy (PBRT), SFRT and SRS. Partial brain radiotherapy was defined as non-stereotactic radiotherapy to only part of the brain. Chart review was performed for all patients who did not complete the prescribed course of radiotherapy, to identify prescribed dose and fractionation schedule. Fractionation was categorized into one to five fractions with conventional dose fractionation (i.e., 20 Gy in five fractions), more than five fractions conventional (i.e., 30 Gy in 10 fractions) and stereotactic radiotherapy (either SRS or SFRT). Systemic therapy delivered prior to radiotherapy was also collected, there were no data on systemic therapy use after radiation. Patients who received more than one course of palliative radiotherapy were considered independently for each course. All treatment courses were used in the analysis, as opposed to individual patients since the investigated outcome is mortality after radiation which reflects the decision-making process for each course.

Associations between patient/treatment characteristics and death within 90 days were assessed using chi-square and t-tests for categorical and continuous variables, respectively. Kendall’s tau was used to assess the correlation between factors. A multicollinearity analyses was performed prior to the multi-variable logistic regression analysis. Factors assessed in both Kendall’s tau and the multicollinearity analysis were age, treatment centre, performance status, histology, radiation technique, fractionation, retreatment, EGFR status, and initial systemic therapy. The thresholds used in this analysis were a variance inflation factor (VIF) of 2.5 and a tolerance level of 0.4. Based on correlation analysis and multicollinearity, radiation technique was excluded from the multivariable analyses since this was collinear with fractionation.

Multi-variable logistic regression analysis was subsequently performed to assess associations. P-values were two-sided and were considered statistically significant when the value was less than 0.05. Analyses were conducted using SPSS version 14.0 software (Chicago, IL). This study was approved by the joint University of British Columbia and BC Cancer Agency Research Ethics Board.

## Results

In total, 740 patients with brain metastases from lung cancer received 826 courses of radiotherapy to the brain (whole brain, partial brain or stereotactic radiotherapy). Sixty patients (8%) received more than one course of brain radiotherapy with a maximum of five courses. Median age at treatment was 65 years (range 32-95 years). Median survival from the first day of radiotherapy was 4.4 months (95% confidence interval (CI) was 4.0-4.8 months). Further patient and treatment characteristics can be found in Table 1.

**Table 1:** Patient and treatment characteristics NSCLC: non-small cell lung cancer; NOS: not otherwise specified; EGFR: epidermal growth factor receptor; ECOG: eastern cooperative oncology group; BC: British Columbia; RT: radiotherapy.

Patient Characteristics		Patients (n=740)
Gender	Male	45%
	Female	55%
Age groups	<50	5%
	50 - <60	25%
	60 - <70	40%
	70 - <80	25%
	≥80	6%
Histology	Adenocarcinoma	58%
	Squamous cell carcinoma	7%
	NSCLC NOS	11%
	Small cell carcinoma	18%
	Other	7%
Sensitizing EGFR mutation	Yes	9%
	No	91%
Treatment Characteristics		Courses of RT to the brain (n=826)
ECOG performance status	0 - 1	44%
	≥2	30%
	Unknown	25%
Initial systemic therapy	Yes	48%
	No	52%
BC Cancer Centre	Vancouver	38%
	Abbotsford	10%
	Prince George	6%
	Surrey	14%
	Kelowna	18%
	Victoria	15%
Completed intended course of RT	Yes	95%
	No	5%
Number of fractions prescribed	1	10%
	2 - 5	66%
	6 - 10	24%
	>10	1%
Radiation dose	20 Gy in 5 fractions	57%
	30 Gy in 10 fractions	20%
	20 Gy in 8 fractions	2%
	11.5-30 Gy in 1 fraction	10%
	25-35 Gy in 3-5 fractions	1%
	Other	11%

WBRT was given in the majority of cases (86%, n=713). Of the remaining 113 cases, 77% (n=89) received stereotactic treatment with either SRS or SFRT. 

Table 2 shows the association between investigated factors and death within 90 days after the start of brain radiotherapy. This table shows that all factors but EGFR mutation are significantly associated with death within 90 days after the start of radiotherapy. Median survival for patients with and without initial chemotherapy was 6.0 (95% CI 5.1 - 6.9) and 2.8 (95% CI 2.4 - 3.3) months, respectively.

**Table 2:** Characteristics associated with 90-day mortality ECOG: eastern cooperative oncology group; BC: British Columbia; NSCLC: non-small cell lung cancer; NOS: not otherwise specified; EGFR: epidermal growth factor receptor; RT: radiotherapy; 3DCRT: 3D conformal radiotherapy; IMRT: intensity modulated radiotherapy.

Characteristic		Proportion who died within 90 days of starting RT	P value
Overall (n=826)		40% (n=330)	
Gender	Male (n=366)	45% (n=163)	0.016
	Female (n=460)	36% (n=167)	
Age groups	<50 (n=38)	26% (n=10)	<0.001
	50 - <60 (n=209)	29% (n=60)	
	60 - <70 (n=327)	39% (n=126)	
	70 - <80 (n=204)	51% (n=103)	
	≥80 (n=48)	65% (n=31)	
ECOG Performance Status	0 - 1 (n=366)	30% (n=110)	<0.001
	≥2 (n=250)	50% (n=126)	
	Unknown (n=210)	45% (n=94)	
BC Cancer Centre	Vancouver (n=310)	31% (n=97)	0.001
	Abbotsford (n=79)	34% (n=27)	
	Surrey (n=112)	48% (n=54)	
	Prince George (n=53)	43% (n=23)	
	Kelowna (n=151)	48% (n=73)	
	Victoria (n=121)	46% (n=56)	
Histology	Adenocarcinoma (n=488)	37% (n=178)	0.001
	Squamous cell ca (n=61)	57% (n=35)	
	NSCLC NOS (n=83)	41% (n=34)	
	Small cell (n=141)	37% (n=52)	
	Other (n=53)	59% (n=31)	
Sensitizing EGFR mutation	Yes (n=70)	34% (n=24)	0.31
	No (n=756)	41% (n=306)	
Prior systemic therapy	Yes (n=396)	27% (n=108)	<0.001
	No (n=430)	52% (n=222)	
Completed intended course of RT	Yes (n=785)	38% (n=295)	<0.001
	No (n=41)	85% (n=35)	
Number of fractions	1-5 fractions conventional (n=538)	52% (n=279)	<0.001
	>5 fractions conventional (n=199)	19% (n=38)	
	SRS/SFRT (n=89)	15% (n=13)	
RT technique	1-2 fields (n=722)	43% (n=313)	<0.001
	≥3 fields 3DCRT/IMRT (n=15)	27% (n=4)	
	Stereotactic (n=89)	15% (n=13)	
Retreatment	Yes (n=59)	17% (n=10)	<0.001
	No (n=767)	42% (n=320)	

The multivariable analysis for death within 90 days after the start of radiotherapy is shown in Table 3. Variables associated with increased odds for death within 90 days of treatment are age, treatment centre and squamous cell carcinoma. Variables associated with decreased odds for death within 90 days of radiotherapy are initial systemic therapy, treatment with more than five fractions and stereotactic radiation.

**Table 3:** Multivariable analyses 90-day mortality CI: confidence interval; ECOG: eastern cooperative oncology group; BC: British Columbia; NSCLC: non-small cell lung cancer; NOS: not otherwise specified; EGFR: epidermal growth factor receptor; RT: radiotherapy; 3DCRT: 3D conformal radiotherapy; IMRT: intensity modulated radiotherapy.

Characteristic		Death within 90 days after start of radiotherapy		
		Odds ratio	95% CI	P-value
Age (continuous)		1.04	1.02 to 1.05	<0.001
Gender	Male	Reference		
	Female	0.82	0.60 to 1.12	0.22
ECOG Performance	0 - 1	Reference		
	≥ 2	1.59	1.09 to 2.31	0.02
	Unknown	1.41	0.94 to 2.10	0.09
BC Cancer Centre	Vancouver	Reference		
	Abbotsford	1.15	0.63 to 2.13	0.65
	Surrey	1.32	0.80 to 2.19	0.29
	Prince George	1.39	0.69 to 2.77	0.35
	Kelowna	1.65	1.04 to 2.61	0.04
	Victoria	1.03	0.62 to 1.71	0.90
Histology	Adenocarcinoma	Reference		
	Squamous cell carcinoma	2.10	1.13 to 3.90	0.02
	NSCLC NOS	1.08	0.64 to 1.85	0.77
	Small cell lung cancer	1.00	0.63 to 1.58	0.99
	Other	1.50	0.76 to 2.90	0.23
Initial systemic therapy	No	Reference		
	Yes	0.48	0.34 to 0.68	<0.001
Fractionation	1-5 fractions conventional	Reference		
	>5 fractions conventional	0.25	0.16 to 0.39	<0.001
	SRS/SFRT	0.29	0.13 to 0.65	0.003
Retreatment	No	Reference		
	Yes	0.77	0.31 to 1.91	0.57
EGFR status	Negative	Reference		
	Positive	1.35	0.76 to 2.43	0.36

## Discussion

This population-based study demonstrates that between 2014-2015 the majority of lung cancer patients in BC, Canada, were treated with WBRT for brain metastases (86% of all treatment courses) and 40% of patients die within 90 days of start of radiation. This is an alarming rate given that brain radiotherapy, especially WBRT, can be associated with considerable side-effects and that the addition of WBRT to best supportive care may not significantly increase survival or improve quality of life [4,10]. Our findings suggest that at a provincial level, a re-evaluation of indications for WBRT and stereotactic treatment for patients with lung cancer is required. Based on our results, we believe that a decrease in the delivery of brain radiotherapy, especially WBRT, is warranted for lung cancer patients who are less likely to benefit from this treatment. To the best of our knowledge, this is the first population-based analysis of the utilization of radiotherapy in lung cancer patients with brain metastases.

More than ¾ of patients received WBRT which is consistent with our initial hypothesis. This high rate is likely explained by the limited access to SRT in the province during our study period, with only 11% of patients receiving this treatment technique. Prior to 2018, SRT has primarily been offered to patients with less than four brain metastases, a good performance status, limited extracranial disease, and a life expectancy of greater than one year based on the Radiation Therapy Oncology Group (RTOG) 9508 trial [15]. However, the increasing life expectancy of patients with stage IV lung cancer secondary to improving systemic therapy has increased the demand for this resource-intensive treatment technique [16-19]. Additionally, phase III trials have consistently identified a superior quality of life and neurocognitive function with SRT compared to WBRT [3,20], and a prospective study of lung cancer patients with up to 10 brain metastases had excellent overall survival and toxicity rates with SRT [21].

The mortality rate 90 days post radiotherapy was 40% in our population, which is higher than we hypothesized although it is consistent with mortality rates found in the literature [22-23]. Our logistic regression analysis identified higher odds for mortality in patients with advanced age, ECOG performance status ≥2 and squamous cell carcinoma. Lower odds were found in patients who received initial systemic therapy and were treated with either SRT or more than five fractions of conventional radiotherapy. These findings are complementary to the RPA and GPA-mol which describe a limited survival for patients in RPA class III and/or GPA-mol score of ≤1.0. The QUARTZ trial would suggest that these patients do not benefit from WBRT [4]. Unfortunately, our mortality rate post WBRT, combined with the high proportion of patients with a poor performance status (ECOG 0-1 in only 44% of patients) and advanced age (31% over 70) suggest that a significant proportion of patients may have been offered potentially futile WBRT near the end of life. This treatment may have been preventable with the consistent application of prognostic tools like the RPA and GPA-mol.

This study should be interpreted in the context of its strengths and limitations. First, our study only captured patients who received radiotherapy, therefore, we cannot estimate how many patients with brain metastases were treated with best supportive care rather than radiotherapy. We also did not assess patients who received systemic therapy alone for their metastatic disease. Variables associated with RPA and GPA-mol, such as extracranial metastases, primary tumor control and number of brain metastases were not collected in this large retrospective review. Karnofsky performance status was not documented in the medical record. It was therefore not possible to apply the RPA or GPA-mol to our study cohort to determine whether or not, based on available evidence, either SRT or best supportive care would have been an appropriate option for the large number of patients who received WBRT. The absence of this information also limits our ability to compare our study population to those in the literature. In this retrospective review, it was also not possible to reliably assess and compare the toxicity and quality of life associated with the different treatment regimes. In our analysis, EGFR status was not associated with survival, which is not consistent with previous studies [17,24], and likely due to the small number of EGFR positive patients in this study.

One of the strengths of this paper is the departmental description of the rate of SRT and WBRT use, which has not been published in the literature yet. This provides a benchmark of radiotherapy utilization for brain metastases in lung cancer patients, to which other departments can compare their use of brain radiotherapy and to which future management changes can be compared. Additionally, this population-based study has a large sample size and is reflective of the lung cancer population with brain metastases as BC Cancer is the sole provider of radiotherapy in the province. It is therefore relatively free from referral and selection bias.

Our findings combined with recent literature have highlighted the need to decrease the utilization of WBRT in our province. Patients with advanced age, poor performance, and short predicted life expectancy based on RPA or GPA-mol, should receive best supportive care rather than WBRT. Patients with a predicted life expectancy of more than three to six months, and up to 10 brain metastases that are small in volume, should be considered for SRT rather than WBRT. This paper provides a useful benchmark for RT usage for lung cancer patients with brain metastases, as changes to our clinical practice guidelines are implemented. It will be important to continually review our practice patterns as management paradigms evolve according to advancing radiotherapy techniques and growing interest in treatment with systemic therapy alone.

## Conclusions

This population-based study identifies that 86% of radiotherapy courses for brain metastases from lung cancer are WBRT and 40% of patients received treatment in the last 90 days of their life. Older age, ECOG performance status ≥2, squamous cell carcinoma histology, and no receipt of systemic therapy were associated with shortened survival. The application of these factors in addition to already established prognostic tools like RPA and GPA-mol should be utilized to facilitate clinical decision making for patients likely to benefit from best supportive care, WBRT or stereotactic treatment. In doing this, WBRT utilization should drastically be decreased, providing improved individualized treatment for patients with brain metastases from lung cancer.

## References

[1] Barnholtz-Sloan JS, Sloan AE, Davis FG, Vigneau FD, Lai P, Sawaya RE (2004). Incidence proportions of brain metastases in patients diagnosed (1973 to 2001) in the Metropolitan Detroit Cancer Surveillance System. J Clin Oncol.

[2] Schouten LJ, Rutten J, Huveneers HAM, Twijnstra A (2002). Incidence of brain metastases in a cohort of patients with carcinoma of the breast, colon, kidney, and lung and melanoma. Cancer.

[3] Wong E, Zhang L, Rowbottom L (2016). Symptoms and quality of life in patients with brain metastases receiving whole-brain radiation therapy. Support Care Cancer.

[4] Mulvenna P, Nankivell M, Barton R (2016). Dexamethasone and supportive care with or without whole brain radiotherapy in treating patients with non-small cell lung cancer with brain metastases unsuitable for resection or stereotactic radiotherapy (QUARTZ): results from a phase 3, non-inferiority, randomised trial. Lancet.

[5] Gaspar L, Mehta M, Patchell R (2010). The role of whole brain radiation therapy in the management of newly diagnosed brain metastases: a systematic review and evidence-based clinical practice guideline. J Neurooncol.

[6] Tsao M, Xu W, Sahgal A (2012). A meta‐analysis evaluating stereotactic radiosurgery, whole‐brain radiotherapy, or both for patients presenting with a limited number of brain metastases. Cancer.

[7] Soliman H, Das S, Larson DA, Sahgal A (2016). Stereotactic radiosurgery (SRS) in the modern management of patients with brain metastases. Oncotarget.

[8] Sahgal A, Ruschin M, Ma L, Verbakel W, Larson D, Brown PD (2017). Stereotactic radiosurgery alone for multiple brain metastases? A review of clinical and technical issues. Neuro Oncol.

[9] Tsao MN, Xu W, Wong RK (2018). Whole brain radiotherapy for the treatment of newly diagnosed multiple brain metastases. Cochrane Database Syst Rev.

[10] Jeene PM, de Vries KC, van Nes (2018). Survival after whole brain radiotherapy for brain metastases from lung cancer and breast cancer is poor in 6325 Dutch patients treated between 2000 and 2014. Acta Oncologica.

[11] Gaspar L, Scott C, Rotman M (1997). Recursive partitioning analysis (RPA) of prognostic factors in three radiation therapy oncology group (RTOG) brain metastases trials. Int J Radiat Oncol Biol Phys.

[12] Sperduto PW, Yang TJ, Beal K (2017). Estimating survival in patients with lung cancer and brain metastases: an update of the graded prognostic assessment for lung cancer using molecular markers (lung-molGPA). JAMA Oncol.

[13] Nagtegaal S, Claes A, Suijkerbuijk K, Schramel F, Snijders T, Verhoeff J (2019). Comparing survival predicted by the diagnosis-specific Graded Prognostic Assessment (DS-GPA) to actual survival in patients with 1-10 brain metastases treated with stereotactic radiosurgery. Radiother Oncol.

[14] Hendriks LEL, Troost EGC, Steward A (2014). Patient selection for whole brain radiotherapy (WBRT) in a large lung cancer cohort: impact of a new Dutch guideline on brain metastases. Acta Oncologica.

[15] Andrews DW, Scott CB, Sperduto PW (2004). Whole brain radiation therapy with or without stereotactic radiosurgery boost for patients with one to three brain metastases: phase III results of the RTOG 9508 randomised trial. Lancet.

[16] Camidge DR, Kim D, Kim HR (2018). Brigatinib versus crizotinib in ALK-positive non-small-cell lung cancer. N Engl J Med.

[17] Eichler AF, Kahle KT, Wang DL (2010). EGFR mutation status and survival after diagnosis of brain metastasis in nonsmall cell lung cancer. Neuro Oncol.

[18] Herbst RS, Baas P, Kim DW (2015). Pembrolizumab versus docetaxel for previously treated, PD-L1-positive, advanced non-small-cell lung cancer (KEYNOTE-010): a randomised controlled trial. Lancet.

[19] Soria J, Ohe Y, Vansteenkiste J (2018). Osimertinib in untreated EGFR-mutated advanced non-small-cell lung cancer. N Engl J Med.

[20] Peters S, Bexelius C, Munk V, Leighl N (2016). The impact of brain metastasis on quality of life, resource utilization and survival in patients with non-small-cell lung cancer. Cancer Treat Rev.

[21] Yamamoto M, Higuchi Y, Sato Y, Aiyama H, Kasuya H, Barfod B (2019). Stereotactic radiosurgery for patients with 10 or more brain metastases. Prog Neurol Surg.

[22] Komaki R, Scott CB, Byhardt R (1998). Failure patterns by prognostic group determined by recursive partitioning analysis (RPA) of 1547 patients on four radiation therapy oncology group (RTOG) studies in inoperable nonsmall-cell lung cancer (NSCLC). Int J Radiat Oncol Biol Phys.

[23] Ryoo JJ, Batech M, Zheng C (2017). Radiotherapy for brain metastases near the end of life in an integrated health care system. Ann Palliat Med.

[24] Yang W, Yang P, Yang JC (2018). Epidermal growth factor receptor mutation predicts favorable outcomes in non-small cell lung cancer patients with brain metastases treated with stereotactic radiosurgery. Radiother Oncol.

